# Clinical-Radiological Outcomes of Early Versus Delayed Weight-Bearing Following Proximal Femoral Nail Antirotation 2 Fixation for Intertrochanteric Fractures: A Prospective Randomized Comparative Study

**DOI:** 10.7759/cureus.101530

**Published:** 2026-01-14

**Authors:** Anup Kumar, Rupendra Jhariya, Sudeep Kumar, Ritesh Pandey, Shikhar Yadav

**Affiliations:** 1 Orthopaedics, All India Institute of Medical Sciences, Patna, Patna, IND

**Keywords:** delayed weight-bearing, early weight-bearing, hip fracture rehabilitation, intertrochanteric fractures, modified harris hip score, pfna2, weight-bearing protocols

## Abstract

Background

Intertrochanteric fractures commonly occur in elderly patients following minor trauma due to osteoporosis, arthritic hip changes, and balance disorders. Early mobilization is crucial to prevent complications of prolonged bed rest. This study compared Modified Harris Hip Scores (mHHS) between early and delayed weight-bearing protocols following proximal femoral nail antirotation 2 (PFNA2) fixation.

Methodology

This prospective randomized comparative study was conducted at a tertiary care center, where patients with intertrochanteric fractures treated with PFNA2 were randomly allocated before surgery into the following two groups: Group 1 (early weight-bearing within 24 hours post-surgery) and Group 2 (delayed weight-bearing after four weeks). Outcomes were assessed using mHHS and radiographs over 24 weeks.

Results

Of the 55 enrolled patients, 46 completed follow-up (21 in the early weight-bearing group, 25 in the delayed weight-bearing group; nine were lost to follow-up). At 24 weeks, mean mHHS was 82.28 ± 12.29 in the delayed group versus 84.95 ± 15.82 in the early group (p = 0.523). The tip-apex distance was 18.13 ± 4.04 mm versus 22.11 ± 9.68 mm (p = 0.068), and Parker’s ratio was 0.56 ± 0.10 in both groups (p = 0.90).

Conclusions

Functional improvement and failure risk are independent of postoperative weight-bearing protocols. Early weight-bearing appears safe and effective, potentially facilitating faster patient mobilization without compromising outcomes. No statistically significant differences were found in mHHS between groups, indicating similar functional recovery. Radiological parameters (tip-apex distance and Parker’s ratio) also showed no significant differences at union.

## Introduction

Intertrochanteric fractures are encountered in both young and elderly populations, but occur more commonly in older age groups. According to the International Osteoporosis Foundation, six million such fractures could occur globally by 2050, up from the current estimate of 1.6 million each year [1]. A 2019 study by Ayoade et al. [2] reported that primary diagnoses of intertrochanteric fractures and all hip fractures had incidence rates per 100,000 of 171 and 402, respectively. Treatment of these fractures is almost always an operative intervention with an extramedullary or intramedullary implant. Postoperatively, patients can be mobilized either with delayed or immediate weight-bearing with support.

Delaying weight-bearing reduces mechanical failure but increases complications related to patient immobilization, such as deep venous thrombosis and pressure sores. Moreover, restricted weight-bearing has been associated with low revision rates (2.9%) after primary fixation of intertrochanteric fractures [3]. Early mobilization counters complications that can occur due to decumbency at old age, but poses the risk of mechanical failure due to implant cut-out secondary to osteoporosis and/or excessive mobilization in this age group. In addition to having a major effect on short-term outcomes such as fewer complications and shorter hospital stays, early mobilization recovery also improves long-term outcomes such as increased autonomy and decreased mortality [4-7]. The goal of surgery is mainly stable fixation, allowing mobilization with early full weight-bearing and rapid return to pre-fracture activity level.

The present study was conducted to determine whether there was any difference in functional recovery as measured by the Modified Harris Hip Score (mHHS) of patients undergoing early weight-bearing compared to late weight-bearing postoperatively in patients undergoing fixation with proximal femoral nail antirotation 2 (PFNA2) for intertrochanteric fractures.

## Materials and methods

Study design and setting

In this prospective study, we analyzed 55 consecutive patients who had intertrochanteric fractures and underwent fixation with PFNA2. This study was conducted in the Department of Orthopedics at a tertiary care center over a defined study period. This was a prospective comparative study utilizing a randomized cohort allocation with two different postoperative weight-bearing protocols.

Ethical approval

Before the commencement of the study, ethical approval was obtained from the Institutional Ethics Committee, All India Institute of Medical Sciences, Patna (approval number: AIIMS/Pat/IEC/PGTh/Jan21/18). Consecutive patients meeting the inclusion criteria were enrolled in the study after providing informed consent.

Inclusion and exclusion criteria

Patients with intertrochanteric fractures type AO31A1, 31A2, and 31A3; those with closed intertrochanteric fractures; those aged 18 years and above; and those who provided consent to participate in the study were considered for inclusion in the study. Patients with open fractures, pathological fractures, multiple fractures, and polytrauma; those with pathologies of the knee or lower limb; those with intraoperative fractures and undergoing additional intervention; and those with previous surgery on the same site were excluded from the study.

Group allocation and randomization

The study population was divided into the following two groups: Group 1 (early weight-bearing) included patients who were randomly allocated to undergo weight-bearing as able mobilization after 24 hours following fixation of the intertrochanteric fracture with a PFNA2 using a walker or axillary crutches. Group 2 (delayed weight-bearing) included patients who were randomly allocated to undergo weight-bearing mobilization only after four weeks following fixation of the intertrochanteric fracture with a PFNA2 using a walker or axillary crutches.

Patients meeting the inclusion criteria were randomized in a 1:1 ratio to Group 1 or Group 2 using a pre-planned randomization protocol before surgical intervention. The randomization sequence was generated before patient enrollment.

Study procedure

Patients were subjected to routine clinical examination and radiological investigations. Preoperatively, mHHS was calculated by the evaluator to assess the pre-fracture status. Patients were randomly allocated to Group 1 or Group 2 before surgery using the pre-planned randomization protocol. Mobilization of patients was done with walker support as soon as possible, and weight-bearing was allowed according to their randomized study group. The duration of hospital stay postoperatively was recorded.

Postoperative protocol

Patients in the early weight-bearing group were made to bear weight as able and mobilized using walkers within 24 hours of surgery. Patients in the late weight-bearing group were made to sit by the side of the bed or made to walk without bearing weight on the operated limb using walker support. All patients in both groups received the same deep vein thrombosis prophylaxis (enoxaparin 40 mg subcutaneously once daily starting 12 hours postoperatively to 48 hours postoperatively, followed by aspirin 75 mg orally once daily for three weeks).

Outcome assessment

**Figure 1:**
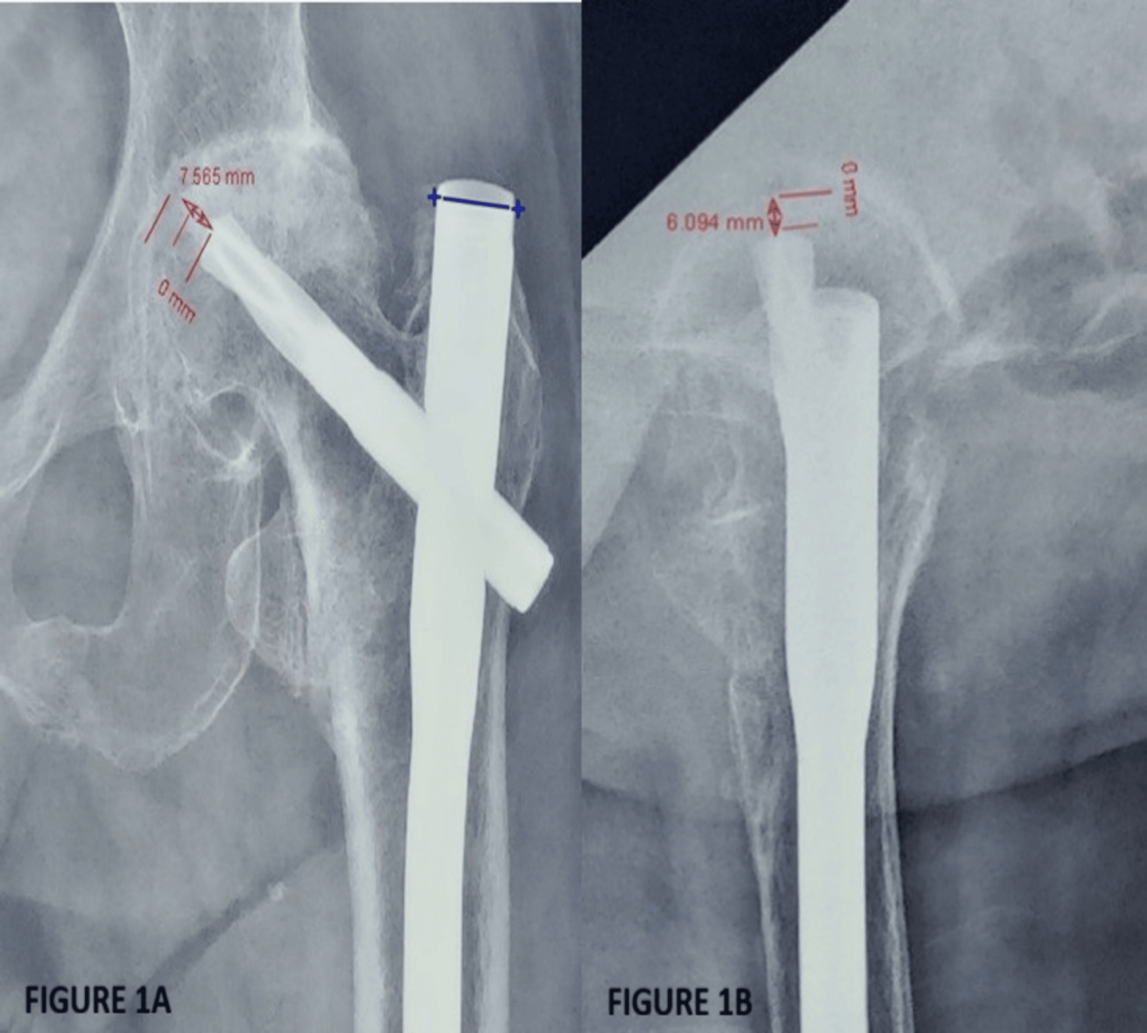
Tip-apex distance calculation on anteroposterior and lateral radiographs. (A) Anteroposterior view. (B) Lateral view. The tip-apex distance is calculated as the sum of measurements in anteroposterior and lateral radiographs. Blue line = reference measurement (proximal nail diameter = 16.5 mm).

**Figure 2:**
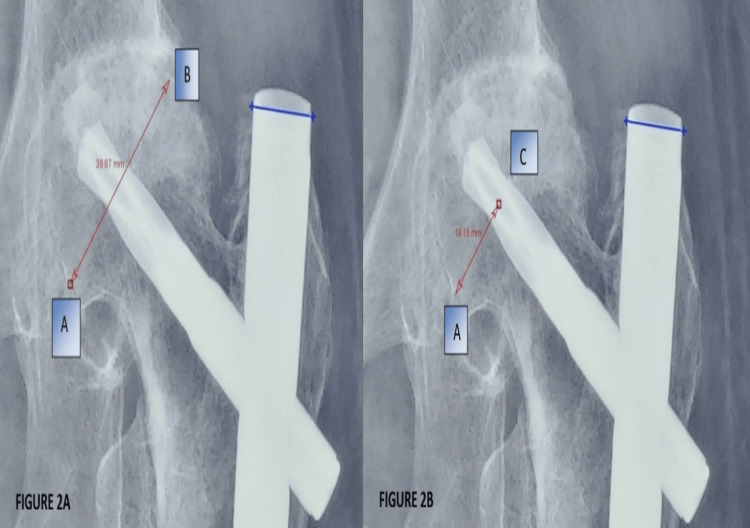
Parker’s ratio calculation demonstrating proper blade positioning. (A) Measurement of the femoral head diameter (Line AB) perpendicular to the femur-neck axis. (B) Perpendicular line to the head-neck axis intersecting at the helical blade mid-point (Line AC). Parker’s ratio = Line AC/Line AB. Blue line = reference measurement.

A comprehensive examination of the hip was performed, and inspection and palpatory findings were documented. To determine functional status using mHHS, patients were provided with scoring sheets, and assistance was provided in filling them as required. Radiological assessment was performed using the tracker app. The tip-apex distance (TAD) and Parker’s ratio were calculated for all radiographs, as shown in Figure 1 and Figure 2. During the measurement, the diameter of the helical blade and the proximal portion of the nail, which were 10.6 mm and 16.5 mm, respectively, were taken as the reference scale. The TAD was calculated from the center of the tip of the helical blade to the center of the femur head in both anteroposterior and lateral views of the radiograph, and then both were summed. Radiographs were also checked for signs of union and complications such as helical blade cutout and implant breakage. Each patient was assessed preoperatively and at 4, 8, 12, and 24 weeks postoperatively.

Statistical analysis

Significance of the association between two categorical variables was assessed using the chi-square test, and the significance of the mean difference was ascertained using Student’s t-test. Repeated measures analysis of variance (ANOVA) was applied to determine the change in the mean score within and between study groups over time. A two-sided p-value of less than 0.05 was deemed statistically significant. SPSS version 23 (IBM Corp., Armonk, NY, USA) was used for data analysis.

## Results

Study participants and baseline characteristics

**Figure 3:**
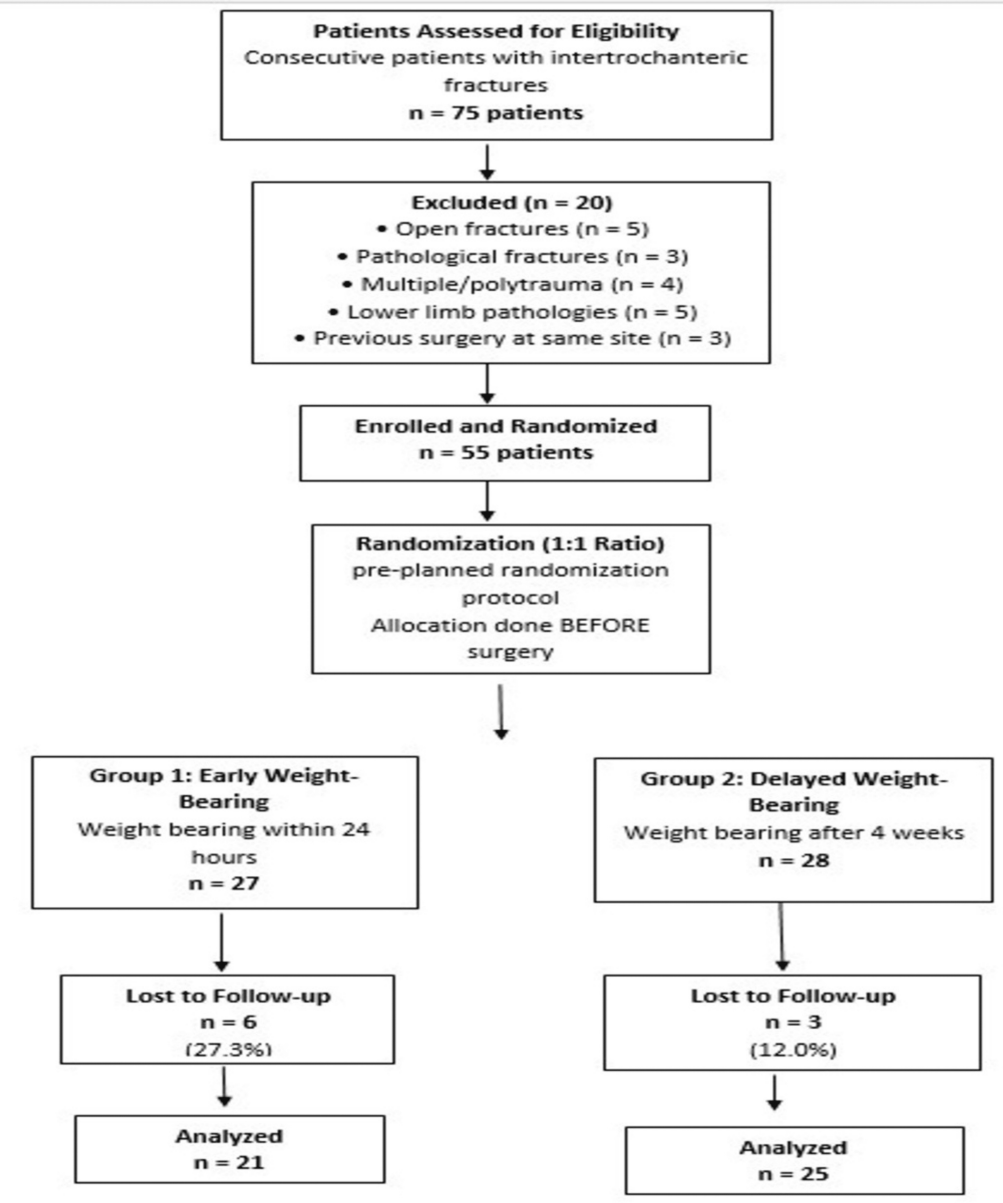
Flow diagram showing patient enrollment, randomization, allocation, follow-up, and analysis. EWB: early weight-bearing; LWB: late/delayed weight-bearing; PFNA2: proximal femoral nail antirotation 2

In this prospective randomized comparative study, patients were randomly allocated into early versus delayed weight-bearing groups before surgical intervention. A total of 55 consecutive eligible patients were randomized to the two groups, though nine patients (six from the early weight-bearing group and three from the late weight-bearing group) were lost to follow-up (Figure 3), leaving 46 patients for the final analysis (21 in the early weight-bearing group, 25 in the late weight-bearing group). The baseline demographic and clinical characteristics of both groups were comparable, with no significant differences in age, gender, fracture side, or fracture classification, indicating successful randomization (Table 1).

**Table 1:** Baseline demographic and clinical characteristics.

Characteristics	Late weight-bearing (n = 25)	Early weight-bearing (n = 21)	P-value
Age (years)	65.80 ± 12.95	69.10 ± 11.67	0.374
Gender			
Male	13 (52.0%)	11 (52.4%)	-
Female	12 (48.0%)	10 (47.6%)	
Fracture side			
Right	16 (64.0%)	14 (66.7%)	-
Left	9 (36.0%)	7 (33.3%)	
Fracture classification			
AO 31A1	15 (60.0%)	14 (66.7%)	-
AO 31A2	7 (28.0%)	5 (23.8%)	
AO 31A3	3 (12.0%)	2 (9.5%)	
Hospital stay (days)	9.1 ± 2.8	8.2 ± 2.1	0.156

Primary outcome: Modified Harris Hip Score

No statistically significant differences were observed in mHHS between the early and delayed weight-bearing groups at any time point throughout the 24-week follow-up period. Both groups demonstrated significant within-group improvement over time (late weight-bearing: F = 168.45, p < 0.001; early weight-bearing: F = 102.92, p < 0.001 on repeated measures ANOVA). Detailed mHHS at each timepoint are presented in Table 2.

**Table 2:** Intergroup comparison of mHHS. Based on the unpaired t-tests and p-values, no significant differences were observed in mHHS between patients who underwent late weight-bearing and those who underwent early weight-bearing at various time points throughout the study. However, there was a significant effect of time within each group, indicating improvement in mHHS over time regardless of the weight-bearing technique employed. mHHS: Modified Harris Hip Score; ANOVA: analysis of variance

mHHS	Late weight-bearing		Early weight-bearing		Unpaired t-test	
	Mean	SD	Mean	SD	t-value	P-value
Baseline	93.16	10.10	96.00	5.18	1.17	0.250
4^th^ week	47.48	9.85	51.67	9.70	1.45	0.155
8^th^ week	56.28	10.68	59.67	12.52	0.99	0.328
12^th^ week	70.80	10.54	73.90	13.64	0.87	0.389
24^th^ week	82.28	12.29	84.95	15.82	0.64	0.523
Intragroup (repeated measures ANOVA)	F = 168.45, p < 0.001		F = 102.92, p < 0.001			

Secondary outcomes: radiological parameters

TAD and Parker's ratio remained comparable between both groups throughout the follow-up period with no significant between-group differences. Detailed radiological measurements at each timepoint are presented in Table 3 and Table 4. No significant differences were observed in postoperative complications, including surgical site infections, deep vein thrombosis, pressure sores, or implant failures, between the two groups during the 24-week follow-up period.

**Table 3:** Intergroup comparison of TAD. Based on the unpaired t-tests and p-values, there were no significant differences in TAD between subjects who underwent late weight-bearing and those who underwent early weight-bearing at the 4th, 8th, 12th, and 24th weeks. TAD: tip-apex distance; ANOVA: analysis of variance

TAD (in mm)	Late weight-bearing		Early weight-bearing		Unpaired t-test	
	Mean	SD	Mean	SD	t-value	P-value
4^th^ week	18.07	4.03	19.54	6.24	0.97	0.339
8^th^ week	17.78	3.76	19.71	4.56	1.58	0.121
12^th^ week	17.92	3.67	20.23	5.49	1.70	0.097
24^th^ week	18.13	4.04	22.11	9.68	1.87	0.068
Intragroup (repeated measures ANOVA)	F = 1.64, p = 0.188		F = 1.39, p = 0.254			

**Table 4:** Intergroup comparison of Parker’s ratio. Based on the unpaired t-tests and p-values, there were no significant differences in the Parker’s ratio between patients who underwent late weight-bearing and those who underwent early weight-bearing at the 4th, 8th, 12th, and 24th weeks. Furthermore, while there was a marginal effect of time on the Parker’s ratio for the late weight-bearing group, the effect size was small and not significant for the early weight bearing group, as determined by repeated measures ANOVA. ANOVA: analysis of variance

Parker’s ratio	Late weight-bearing		Early weight-bearing		Unpaired t-test	
	Mean	SD	Mean	SD	t-value	P-value
4^th^ week	0.54	0.08	0.53	0.08	-0.40	0.693
8^th^ week	0.54	0.09	0.55	0.07	0.45	0.656
12^th^ week	0.54	0.08	0.55	0.07	0.37	0.714
24^th^ week	0.56	0.10	0.56	0.10	0.13	0.900
Intragroup (repeated measures ANOVA)	F = 2.74, p = 0.050		F = 1.60, p = 0.200			

Functional outcome at 24 weeks

At the end of 24 weeks, functional assessment demonstrated excellent or good outcomes in 69.5% of patients (20 with excellent outcomes, 12 with good outcomes). Fair outcomes were observed in 17.39% (n = 8), while poor outcomes occurred in 13.04% (n = 6) of participants (Table 5).

**Table 5:** Functional assessment using mHHS grade at the end of 24 weeks. mHHS: Modified Harris Hip Score

mHHS	Number of patients	Percentage (%)
Excellent	20	43.4
Good	12	26.08
Fair	8	17.39
Poor	6	13.04

Subgroup analyses

Subgroup analyses stratified by age groups (≤65 years vs. >65 years), fracture type (31A1 vs. 31A2/A3), and gender demonstrated consistent findings with no significant between-group differences in mHHS outcomes, suggesting that early weight-bearing is safe and effective across diverse patient populations.

## Discussion

Main findings

Hip fractures can change the course of an orthogeriatric patient’s life. Reduction of mobility in hip fracture patients poses a significant threat to their clinical outcome, quality of life, and survival, particularly in the elderly population. Early mobilization without weight-bearing restrictions has been shown to benefit these patients. Conversely, prolonged bed rest and difficulty in mobilization can lead to various complications, including urinary tract infections, respiratory pneumonia, deep vein thrombosis, and pressure sores. Even brief periods of muscle disuse can result in substantial losses in muscle mass and function [8].

The results of this prospective randomized comparative study demonstrate that early weight-bearing does not adversely impact functional outcomes compared to delayed weight-bearing protocols. Both groups showed significant within-group improvement in mHHS over the 24-week follow-up period, with no statistically significant between-group differences. Radiological parameters (TAD and Parker’s ratio) also remained comparable between groups, indicating that implant positioning and stability were not compromised by early mobilization. Additionally, subgroup analyses stratified by age, fracture type, and gender consistently demonstrated comparable outcomes between protocols, suggesting that early weight-bearing is safe and effective across diverse patient populations.

Comparison with existing literature

These findings suggest that early weight-bearing may be a viable option for patients undergoing fixation of intertrochanteric fractures with PFNA2, without compromising functional outcomes. Our results align with Siu et al. [9], who reported that mobilization in the early postoperative phase is of superior importance, as delays are associated with diminished physical function at two months and worse survival rates at six months. Our findings are further supported by Kuru et al. [10], who reported that early weight-bearing within 24 hours postoperatively results in significantly better Harris and pain scores compared to delayed weight-bearing. Similarly, Ottesen et al. [11] found that elderly hip fracture patients with weight-bearing restrictions had higher rates of adverse events, including delirium, infection, and mortality, compared to those allowed to weight-bear as tolerated.

Further, our results are consistent with a retrospective analysis by Takemoto et al. [12] of 293 patients, who found that weight-bearing restriction did not decrease the incidence of surgical failures; moreover, the non-weight-bearing group had significantly more surgical complications than the weight-bearing group. A comparable study by Vishwanathan et al. [13] included 81 patients treated with proximal femoral nail, with 75 patients completing follow-up, and revealed that the mHHS significantly improved at one, three, and six months postoperatively. Another study by Jia et al. [14] conducted among 806 matched patients revealed no significant difference in implant failure at 12 months between immediate and restricted weight-bearing groups; however, patients with immediate weight-bearing had a significantly shorter time to full weight-bearing (87.6 vs. 121.3 days).

A study by Pfeufer et al. [15] reported that elderly hip fracture patients with postoperative weight-bearing restrictions had significantly reduced mobility and gait speed compared to those with full weight-bearing (Parker Mobility Score: 3.21 vs. 4.73, p < 0.001; gait speed: 0.16 m/s vs. 0.28 m/s, p = 0.003). Another similar study by Oldmeadow et al. [4] found that at one-week post-surgery, patients in the early ambulation group walked further than those in the delayed ambulation group (p = 0.03) and required less assistance to transfer (p = 0.009). Compared to the delayed ambulation group, patients in the early ambulation group were less likely to require high-level care (36.8% vs. 56%) and more likely to be sent home straight from acute care (26.3% vs. 2.4%).

A study by Li et al. [16] investigated the effects of varying loads on the PFNA2 implant, specifically examining displacement and loading cycles under different weight-bearing conditions post-implantation. Their findings indicated that in elderly patients with unstable intertrochanteric femoral fractures, the nail offers the primary postoperative structural support and stability; however, weight-bearing should be restricted to 900 N to reduce the risk of implant failure and complications. Therefore, early initiation of graduated weight-bearing is permissible, as long as it remains within the mechanical strength of the implant.

Generalizability and clinical implications

This study was conducted in a single tertiary care center in India and involved predominantly elderly patients with stable intertrochanteric fractures treated with PFNA2 implants. The findings may have limited generalizability to other healthcare settings with different resources, surgical expertise, postoperative rehabilitation protocols, or patient populations with different baseline characteristics and comorbidity profiles. Furthermore, the study population was relatively homogeneous in terms of implant type (PFNA2), and results may not directly apply to other fixation methods, such as the dynamic hip screw or intramedullary nailing. Future multicenter prospective randomized controlled trials are needed to validate these findings across diverse populations and healthcare settings.

Study limitations

This study had several important limitations that warrant consideration. First, although patients were randomly allocated to weight-bearing groups before surgery, this prospective randomized comparative study was designed and reported as an observational comparative study rather than with full randomized controlled trial (RCT) methodology (such as concealment of allocation sequence, blinding of participants/assessors, or pre-registered protocol). This methodological design choice may affect the interpretation of causality compared to a fully reported RCT. However, the randomized allocation before surgery does reduce selection bias from postoperative decision-making based on operative findings.

Second, the surgeries were performed by different surgeons, which may have introduced variations in reduction quality, fixation technique, and implant positioning, potentially affecting radiological parameters (TAD and Parker’s ratio) and functional outcomes. These differences could have masked or exaggerated the true effects of weight-bearing protocols.

Third, our follow-up period was relatively short (24 weeks), precluding assessment of the complete time required for individuals to recover and return to their pre-injury functional status. Long-term outcomes beyond six months, including late implant failures and return to community ambulation, were not evaluated.

Fourth, the study was conducted at a single tertiary care center with a specific patient population, which may limit the generalizability of findings to other healthcare settings, patient demographics, or geographic regions with different resource availability and patient characteristics.

Fifth, loss to follow-up occurred in 16.4% of enrolled patients (9 of 55), with slightly higher attrition in the early weight-bearing group (27.3% vs. 12%). This differential loss could introduce bias if the reasons for dropout were related to weight-bearing status or clinical outcomes.

Sixth, this study was not powered for subgroup analysis by AO classification. Future larger studies should examine whether fracture severity influences weight-bearing protocol outcomes.

Finally, while randomization was performed, potential unmeasured confounding variables such as bone quality assessment, specific comorbid medical conditions, smoking status, medication use (particularly anticoagulants or bisphosphonates), and variations in surgeon experience could have influenced outcomes independent of the weight-bearing protocol itself.

## Conclusions

This prospective randomized comparative study supports early weight-bearing as a safe and effective option for patients with intertrochanteric fractures treated with PFNA2. No significant differences were observed in complication rates, functional recovery, or radiological outcomes between early and delayed weight-bearing groups. These findings align with existing literature, further reinforcing that early mobilization does not compromise fracture healing or fixation stability. Early weight-bearing within 24 hours post-surgery appears to be a viable and safe option that may facilitate faster patient mobilization and potentially reduce complications associated with prolonged immobilization, without compromising functional or radiological outcomes.
